# Evaluation of the Accuracy, Usability, and User Perspectives of the Ecological Momentary Dietary Assessment App Traqq Among Dutch Adolescents: Protocol for a Mixed Methods Study

**DOI:** 10.2196/70194

**Published:** 2025-11-11

**Authors:** Lieke L E Kennes, Desiree A Lucassen, Anouk M M Vaes, Annemarie Wagemakers, Indre Kalinauskaite, Edith J M Feskens, Elske M Brouwer-Brolsma

**Affiliations:** 1 Division of Human Nutrition and Health Department of Agrotechnology and Food Sciences Wageningen University & Research Wageningen The Netherlands; 2 Health and Society Department of Social Sciences Wageningen University & Research Wageningen The Netherlands; 3 Public Health Practice team Department of Global Public Health & Bioethics at Julius Center for Health Sciences and Primary Care University Medical Centre Utrecht Utrecht The Netherlands

**Keywords:** dietary assessment, app, recall, accuracy, usability, evaluation, mixed methods study, technology, adolescents

## Abstract

**Background:**

Self-reported dietary intake data are crucial in nutrition and health research; however, their accuracy is compromised by challenges such as portion size estimations, food identification, memory-related bias, social desirability bias, and reactivity bias. Dietary assessment in adolescents is particularly challenging due to irregular eating habits, meal skipping, and parent or peer influences, potentially resulting in misreporting. Leveraging adolescents’ receptiveness to technology, we investigated the use of an innovative smartphone app (Traqq) that facilitates dietary assessment using repeated short recalls instead of traditional 24-hour recalls. Evaluation studies of the Traqq app in Dutch adults have shown successful results, but its suitability for other target populations, such as adolescents, requires further investigation.

**Objective:**

We designed a comprehensive, 3-phase study to evaluate the Traqq app’s accuracy using repeated short recalls, usability, and user perspectives among Dutch adolescents aged 12 to 18 years. This manuscript details the study setup, research methods, and basic characteristics in phases 1 and 2.

**Methods:**

In phase 1, adolescents (aged 12-18 years) downloaded the Traqq app and completed a demographic questionnaire. It was used on 4 random school days over 4 weeks, using 2-hour recalls on 2 days and 4-hour recalls on 2 days. A food frequency questionnaire and 2 interviewer-administered 24-hour recalls served as dietary reference methods to assess the Traqq app’s accuracy. In addition, usability was evaluated using the System Usability Scale and an experience questionnaire. In phase 2, user experiences were further explored through semistructured interviews within a subsample of 24 adolescents. These first 2 phases of this mixed methods study are now finalized for data collection. Phase 3 will focus on collecting user insights to inform app customization through cocreation sessions.

**Results:**

Recruitment concluded in September 2022 with 102 adolescents; 98 (96%) provided dietary data via the Traqq app, and 79 (78%) completed the evaluation questionnaire. Adolescents had a mean age of 15 (SD 2) years. The mean BMI was 19.9 (SD 3) kg/m^2^. A total of 64 (63%) participants were girls, 81 (84%) attended high school, and 88 (92%) were born in the Netherlands. Interviews were held with 6 (25%) boys and 18 (75%) girls. Cocreation sessions will be planned after all data have been analyzed.

**Conclusions:**

In this holistic study, we combine quantitative and qualitative methods to evaluate the dietary assessment performance among adolescents of the Traqq app, which was initially designed for adults. Specifically, next to quantitative comparisons of the Traqq app’s dietary assessment methods, we conducted semistructured interviews, and we will carry out cocreation sessions. With this user-centered, synergistic approach, we aim to establish a list of requirements for a dietary assessment app for adolescents, resulting in more efficient assessments, improved compliance, and enhanced overall accuracy in this population.

**Trial Registration:**

ISRCTN Registry ISRCTN46230386; https://www.isrctn.com/ISRCTN46230386

**International Registered Report Identifier (IRRID):**

DERR1-10.2196/70194

## Introduction

Accurate dietary assessment is crucial for understanding the relationship between the intake of food, energy, and nutrients and nutrition-related health issues [1-4]; however, this is inherently challenging. Currently, many studies exploring diet-disease associations mainly rely on self-report methods, such as food frequency questionnaires (FFQs), 24-hour recalls (24hRs), and food records, which are prone to bias, for example, memory-related bias, social desirability bias, and measurement errors related to difficulties in estimating portion sizes. Assessing adolescents’ dietary intake is particularly challenging [5,6] due to their irregular eating habits, meal skipping, dining out [7,8], and susceptibility to peer or caregiver influence, which can potentially result in misreporting [9,10]. In addition, adolescents perceive current dietary assessment methods, especially for reporting complex meals [6,10], as challenging, leading to low compliance rates [11]. Adolescents’ receptiveness to technology [5,12], particularly mobile assessment technologies [13], could significantly advance dietary assessment in this challenging population.

Few digital tools are specifically designed for adolescents, and those available often replicate traditional dietary assessment methods such as 24hRs or food records in web or app-based formats [8,14-18]. Adolescents generally prefer apps over web-based technologies, resulting in more complete registration [13]. However, these tools still face common method-related limitations such as estimation errors, errors related to food identification, social desirability bias, and memory-related bias [11,18]. Developers of the EaT app, a food record app for young adults (aged 18-30 years), addressed issues related to food identification by tailoring the food database to adolescents’ dietary habits. They recorded purchase locations, tagged food items to identify outlets, included foods from the 30 largest ready-to-eat food chains in Australia, removed less-common items, simplified food names, and merged nutritionally similar foods. These improvements enhanced the app’s usability, highlighting the need to tailor tools specifically for adolescents to improve compliance [19].

The web-based 24hR tool, myfood24, has also been tailored for use among adolescents [20,21] by including features such as autofill, spell check, and tooltips. While it showed promising reliability compared to interviewer-administered 24hRs, concerns remained about adolescents’ memory during dietary recall [8]. Nevertheless, research shows that adolescents can accurately recall foods consumed within 24 hours [6], with shorter recall windows potentially providing even more accurate data. Therefore, a promising alternative to traditional dietary assessment methods is near real-time recording, based on the ecological momentary assessment principles [22], via repeated short recalls on a single day, such as repeated 2-hour recalls (2hRs) and 4-hour recalls (4hRs). These short time intervals reduce memory reliance, require minimal time commitment, and reminders help users to report their intake throughout the day [23,24]. We previously developed such an ecological momentary dietary assessment app for adults (Traqq), as described elsewhere [25], with validated repeated 2hRs on 1 day, offering more accurate assessments than traditional 24hRs [26]. While the use of short recalls via the Traqq app has shown promise in adults, its application for adolescents still needs to be investigated.

In addition to evaluating the use of short recalls via the Traqq app, the app itself also needs to be evaluated among adolescents. The app’s simple and clear design, effective for adults, may not appeal as much to adolescents. Research emphasizes the importance of engaging features such as game elements, social features, rewards, and motivational messages to encourage adolescents in using a dietary assessment app [11]. Such features are not included in the Traqq app’s adult version. Therefore, it is imperative to assess and adapt the current design of the Traqq app to meet the needs and preferences of adolescents [27] using a combination of both quantitative and qualitative methods.

The objectives of this observational mixed method study are (1) to *quantitatively* evaluate the accuracy of the smartphone-based 2hR and 4hR methods for assessing energy, nutrient, and food group intake in adolescents compared to traditional methods and usability questionnaire, (2) to gather further user perspectives through *qualitative* semistructured interviews, and (3) to obtain a list of requirements facilitating the redesign of Traqq to better meet adolescents’ needs and preferences through cocreation sessions. In this paper, we detail the study setup and research approaches used to address the objectives outlined earlier.

## Methods

### Study Design

**Figure 1:**
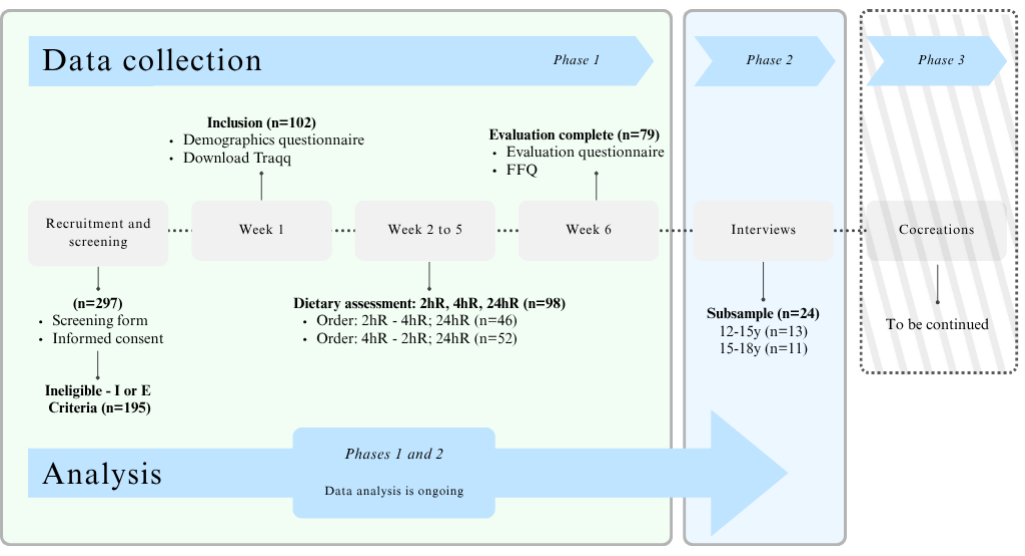
Flowchart of the Traqq-Z study design. 2hR: 2-hour recall; 4hR: 4-hour recall; FFQ: food frequency questionnaire.

In the first phase of this mixed methods study, a quantitative evaluation of the accuracy, usability, and user perspectives of the Traqq app was performed among 102 adolescents. Phase 2 included a qualitative approach to evaluate user experiences. The third and final phase will involve cocreation sessions with 10 to 12 adolescents (Figure 1). Data collection for phases 1 and 2 has been completed, with data analysis currently ongoing. Phase 3, the cocreation sessions, will take place after all data from phases 1 and 2 have been analyzed.

In week 1 of phase 1 (ie, the quantitative evaluation), adolescents downloaded the Traqq app and completed a demographic questionnaire. Over the following 4 weeks, dietary intake was assessed via the Traqq app on 4 random nonconsecutive days, via two 2hR days and two 4hR days. Two interviewer-administered 24hRs and an extensive FFQ at the end of the period served as reference methods. Adolescents also completed an online evaluation questionnaire. In phase 2, semistructured interviews were conducted with a subsample. Phase 3, the cocreation sessions, will be scheduled after analyses of data from phases 1 and 2 have been completed.

### Ethical Considerations

On January 19, 2022, the Medical Ethics Committee (METC Oost-Nederland) concluded that this study did not fall under the Dutch Medical Research Involving Human Subjects Act (2022-13477). Subsequently, on February 23, 2022, this study received approval from the Social Sciences Ethics Committee at Wageningen University & Research. Prior to participation, all participants and their parents or caregivers provided written informed consent. All data were anonymized in accordance with ethical standards, ensuring no personal identifiers were present. Participants received a small gift voucher and a personalized dietary report as a token of appreciation.

### Study Population

Recruitment for phase 1 took place between February 2022 and September 2022 and aimed to include 100 adolescents (ie, 50 adolescents aged 12-15 years and 50 adolescents aged 16-18 years). Adolescents were recruited through the volunteer database of the division of Human Nutrition and Health of Wageningen University & Research and other channels, such as social media and schools in Wageningen and neighboring municipalities (the Netherlands). The volunteer database consists of volunteers aged more than 18 years. Adults born between 1960 and 1985 were notified about the study and asked if their child in the age range of 12 to 18 years would be willing to participate. Adolescents willing to participate were invited to an online information meeting (n=297), with parents or caregivers joining for those aged below 16 years. Before the information meeting, an information letter including an informed consent form was sent. During the meeting, the study was explained and questions were answered. Adolescents aged 16 years and older signed their own consent, while those aged 12 to 15 years provided coconsent with a parent or caregiver. Adolescents were eligible if they spoke and read Dutch, were not visually impaired, had a smartphone with internet and email, were willing to maintain current dietary habits for the duration of the study, and were not participating in a dietary intervention study. A total of 102 adolescents were included. Among them, 53 (52%) adolescents were aged 12 to 15 years and 49 (48%) adolescents were aged 16 to 18 years. After phase 1, 24 adolescents joined the qualitative evaluation in phase 2. Among them, 13 (54%) adolescents were aged 12 to 15 years and 11 (46%) adolescents were aged 16 to 18 years. Phase 3 aims to include 10 to 12 adolescents.

### Phase 1

#### Dietary Assessment

##### Overview

Dietary intake was assessed with the Traqq app on 4 random nonconsecutive days within a 4-week study period, using two 2hR days and two 4hR days. Adolescents were randomly assigned to start with either 2hR days (n=46, 47%) or 4hR days (n=52, 53%). Recalls were scheduled on weekdays for consistency with typical school routines. Interviewer-administered 24hRs and an FFQ served as reference methods, with 24hRs unannounced on nonrecall weekdays. Adolescents completed an FFQ at the end of the study.

##### App-Based 2hR and 4hR Days

**Figure 2:**
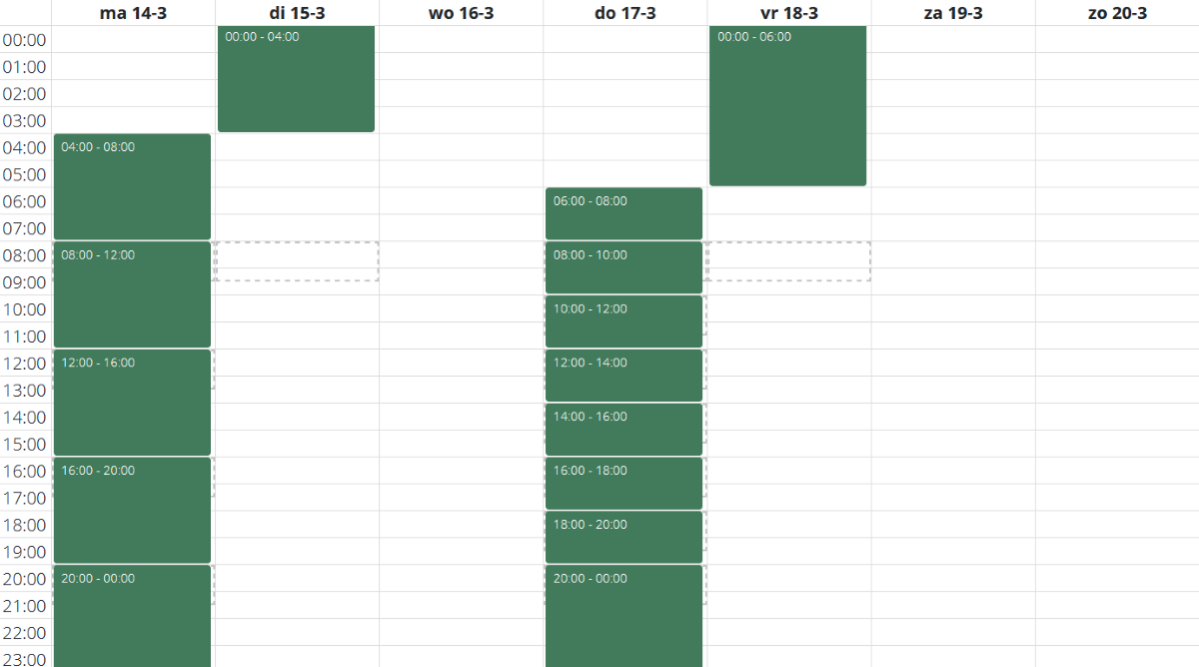
Example of a 2-hour recall day (Monday) and a 4-hour recall day (Thursday).

On 2 random days, adolescents received invitations via the Traqq app to report their food intake every 2 hours (ie, 2hR days), and on 2 other days, every 4 hours (ie, 4hR days). This resulted in 8 reminders on 2hR days and 4 on 4hR days with a reporting window of 90 minutes; for example, for a single 2hR between 6 AM and 8 AM, the notification was sent at 8 AM with a reporting deadline of 9:30 AM. The morning following a Traqq recall day, an invitation was sent to assess nighttime food intake (Figure 2). The 2hR-day and 4hR-day sampling schemes were tailored to each adolescent’s sleeping patterns to avoid disturbances. To illustrate, if an adolescent indicated waking up at 9 AM, the first invitation was sent at 10 AM instead of 8 AM. No invitations were sent after 10 PM. If an adolescent missed more than 1 recall (except nighttime), that day was considered invalid and a new recall day was scheduled.

Adolescents reported their food intake directly in the app by clicking on notifications or opening the Traqq app. The Traqq app uses an extensive food list based on the Dutch Food Composition Database [28]. After selecting a food item, adolescents reported the quantity in household measures (eg, cups or spoons), standard portion sizes (eg, small or large), or grams, along with the eating occasion (ie, breakfast, lunch, dinner, or snack). Adolescents could also use the *My Dishes* option to record ingredients for specific recipes or create quick entries for frequently consumed combinations (eg, daily breakfast). If adolescents did not consume anything during a specified time window, they could select the “I did not eat or drink anything” option [25]. While adolescents were instructed to complete recordings independently, they were advised to ask parental assistance for home-cooked meals, particularly for specific recipes or ingredients, and the app prompted them if help was needed.

##### Interviewer-Administered 24hRs

In addition to the 2hR and 4hR days, 2 unannounced 24hRs were administered via telephone by trained dietitians using the multiple-pass approach [29]. Similar to the Traqq app, portion size estimation methods included household measures, standard portions, or grams. To minimize disruption to school hours, adolescents were contacted in the afternoon or evening, with multiple attempts made to reach them on recall days. In case of nonresponse, a new 24hR was randomly scheduled. Dietitians transcribed 24hRs into food codes using the Dutch Food Composition Database [28]. Regular meetings were held with all dietitians to ensure quality and consistency in food coding.

##### Computation of Dietary Recall Data

Data from the 2hR days, 4hR days, and the 24hRs were entered in the computation module of Compl-eat [30]. Total intakes of energy, macronutrients, micronutrients, and food groups were calculated using the Dutch Food Composition Database [28]. Dietary intake data were thoroughly checked by trained dietitians, according to a standardized protocol. Dietitians checked the data for completeness and unusual amounts. Reporting errors were corrected according to a standardized approach, using standard portion sizes and recipes, for example, 250 glasses of cola was corrected to 1 glass of 250 g. Adolescents were not contacted in case of discrepancies.

##### Web-Based FFQ

At the end of the 4-week study period, adolescents were asked to complete a validated semiquantitative FFQ, with a reference period of 4 weeks [31]. This extensive FFQ was administered online with the self-administered Dutch FFQ tool [32]. Adolescents indicated the frequency of consumed food items by selecting answers ranging from “not consumed” to “7 days per week.” In addition, portion sizes were estimated using natural portions and common household measures. Energy and nutrient contents of foods were based on the Dutch Food Composition Database [33] and multiplied by the portion size and frequency of consumption to calculate the mean daily intake of energy, macronutrients, and micronutrients. In addition, the average daily intake (in grams) of food items was calculated by multiplying the frequency of consumption by portion size. Trained dietitians conducted multiple quality checks to safeguard the quality of the data.

#### Demographics

At the start of the study, adolescents completed a demographic and personal characteristics questionnaire, including questions about the date of administration, sex (“boy” or “girl”), and country of birth (“the Netherlands” or “other”). Age was categorized into 12- to 15-year and 16- to 18-year age groups. Adolescents were asked, “Who do you live with most days of the week?” with 7 options: “both parents or caregivers,” “2 cohabiting parents,” “mother and partner,” “father and partner,” “mother,” “father,” “living independently,” or “other.” For analyses, coparenting, living with the mother or father and partner, and living with the mother or father separately were grouped as living separately. The education level was categorized according to the Dutch system into 3 options: “primary school” (including “grade 8 [±12 years]” and “other”), “secondary school” (including “year 1” to “year 6” and “other”), and “other” (including “Vmbo,” “Vmbo-t [theoretical, mavo],” “Vmbo-(g)t,” “Havo,” “Vwo,” and “other”). For analyses, “Vmbo,” “Vmbo-t,” and “Vmbo-(g)t” were grouped as intermediate vocational education and “Havo” and “Vwo” as higher vocational and academic education. Height and weight were collected to calculate BMI (kg/m²) and categorized into normal weight, underweight, and overweight based on age- and sex-specific BMI cutoffs according to the Schofield 10 to 18 years equation:

- For girls: basal metabolic rate = (200.0 + [8.4 × weight]) + (465.4 × [length / 100]).
- For boys: basal metabolic rate = (515.3 + [16.2 × weight]) + (137.1 × [length / 100]) [34].

Adolescents self-reported their weekly physical activity by indicating how many days (<1 d up to >7 d) they exercised for at least an hour, including all forms of sports or exercise and commuting to school. Other questions about physical activity included sports (“yes” or “no”), such as fitness, swimming, tennis, etc; average number of times exercise was performed per week (<1 time per wk up to >7 times per wk); and average duration of exercise (<1 h up to >2 h). The last 2 questions were converted into 1 variable, “average exercise time.” Questions about whether adolescents follow a diet to gain weight (“yes” or “no”), a diet to lose weight (“yes” or “no”), and their sleeping patterns were also included.

#### User Evaluation

During the final study week, adolescents completed an evaluation questionnaire about their experiences with the dietary assessment methods (ie, 2hR days, 4hR days, 24hRs, and FFQ) and the Traqq app in general [35]. The questionnaire, adapted from previous studies, assessed ease-of-use, convenience, reporting burden, perceived accuracy, likelihood of future use, and overall experience [36,37]. It included a question about parental assistance in home-cooked meals and app use, with responses recorded on a 5-point Likert scale from 1 (strongly disagree) to 5 (strongly agree). Adolescents also completed the System Usability Scale (SUS) for the Traqq app [38], a 10-item questionnaire with a similar 5-point Likert scale from 1 (strongly disagree) to 5 (strongly agree) [35,39,40]. Adaptations were made for clarity within this specific population. The SUS score was calculated by subtracting 1 from the scale position for positive questions and subtracting the scale position from 5 for negative questions. The average was then multiplied by 2.5 to obtain the final overall value (range 0-100) [38]. Out of 100, an SUS score >68 indicates above-average usability, while a score >80 indicates excellent usability [38].

#### Quantitative Analysis

In this paper, demographic characteristics are presented as means with SDs for continuous variables and as frequencies (n and %) for categorical variables in adolescents for phases 1 and 2. Future analysis will evaluate the accuracy of the 2hR and 4hR days methods against 24hRs and the FFQ using correlation coefficients, mean and percent differences, Wilcoxon signed rank, and ANOVA tests [26]. Criteria as specified by Lombard et al [41] will be used as the reference to interpret the results. Usability will be evaluated using descriptive statistics per answer category (number and percentage). Statistical analyses were and will be performed with SPSS software (version 28.0.1.1; IBM) and R Statistical open-source software (version 4.3.3; Hornik and R Core Team).

### Phase 2

#### Semistructured Interviews

Qualitative semistructured interviews were conducted online via Microsoft Teams by a single researcher (LLEK) in September 2022. This approach was chosen for its effectiveness, which is comparable to in-person interviews [42], and aimed to explore adolescents’ experiences and preferences regarding the use of the Traqq app. The interview script (Multimedia Appendix 1) included questions about user-friendliness (ie, “What makes it enjoyable or interesting to use the app?”), intuitiveness (ie, “How did you experience using the app?”), perceived burden (ie, “Were there specific time periods when tracking was more difficult?”), notification timing (ie, “What did you think of the response time for notifications?”), interface usability (ie, “How can the app’s clarity be improved?”), food entry clarity (ie, “How did you experience logging your foods and drinks?”), portion size estimation (ie, “How was your experience estimating portion sizes?”), motivation for long-term use (ie, “How many consecutive days would you like to use the app?”), and preferences for future app use (ie, “What makes it enjoyable or interesting to use the app?”). During each session, adolescents also performed a think-aloud exercise [43,44], verbally expressing their thoughts while reporting their dietary intake for the past 4 hours.

#### Qualitative Analysis

Semistructured interviews (length between 22 and 49 min) were audio-recorded and transcribed verbatim. The capability, opportunity, motivation-behavior (COM-B) model was used to inform the subsequent content analysis [45]. This model suggests that for individuals to perform a specific behavior (eg, using an app), they must possess the necessary capabilities (ie, physical and psychological ability to use of the Traqq app), have adequate opportunities (ie, physical and social environment that facilitate the use of the Traqq app), and be sufficiently motivated (ie, automatic and reflective mechanisms that activate or inhibit the use of the Traqq app) [45,46]. Comprehensive analysis was achieved by using a combination of deductive and inductive coding methods. The deductive coding was guided by the COM-B model, which provided a theoretical framework to categorize behavioral determinants. Inductive coding allowed for the identification of additional themes emerging from the data that may not be captured within the COM-B structure [45,46]. Both researchers independently coded all transcripts, and any differences in code lists were discussed and adjusted to reach consensus, resulting in a final list. To ensure accessibility, all codes were translated into English, and the coding and analysis of the interviews were conducted using QDA Miner Lite (version 2.0.9; Provalis Research). Further analysis of the semistructured interviews is ongoing.

### Phase 3

#### Cocreation

During the third phase of the study, a cocreation approach will be used to redesign the Traqq app’s interface, actively involving end users to better align with adolescents’ needs and preferences. At least 2 cocreation sessions will take place, each with approximately 5 adolescents per session, divided by each age group (ie, 12-15 years and 16-18 years age groups). Insights from the semistructured interviews will inform these sessions.

Each session starts with an icebreaker exercise, followed by discussions on user personas and experience journey maps derived from the interview analyses [47,48]. First, adolescents will review identified touchpoints—interaction moments with the Traqq app—and reflect on the emotions associated with them, particularly those eliciting negative emotions. In the second part of the cocreation session, adolescents will collaborate in groups to redesign interactions with the Traqq app that mitigate these negative emotions, using paper prototyping tools. Both groups will test each other’s prototypes, enhancing the codesign process quality, engagement, and educational value. In this case, each group could use the other group to “user test” their paper prototypes.

#### Qualitative Analysis

The researchers will collect, process, and analyze all data. Because this project follows a cocreation and coanalysis approach, not all procedures need transcription, as adolescents will document many aspects. However, key parts of the recordings from the sessions will be transcribed. Data analysis will involve coding by 2 researchers using Atlas.ti/QDA Miner Lite, using a combination of general inductive and deductive thematic analysis [49]. Data collection and analysis will be conducted in a forthcoming phase of the study.

## Results

### Participant Characteristics (Phase 1)

In total, 102 participants were included in phase 1 of the Traqq-Z study (Table 1). The mean age was 15 (SD 2) years. Most (n=64, 63%) of the participants were girls. A total of 88 (92%) participants were born in the Netherlands. Most (n=81, 85%) of them attended high school and 36 (37%) engaged in exercise for an average of more than 6 hours per week. A total of 40 (42%) participants were physically active for an average of 3 to 5 days per week. Moreover, 84 (88%) participants had a healthy BMI, and 75 (78%) lived with both parents. The mean SUS score was 72 (SD 15).

**Table 1:** General characteristics of the adolescents included in phase 1 of this study (N=102).

		Total^a^	12- to 15-year age group	16- to 18-year age group
Sample, n (%)		102 (100)	53 (52)	49 (48)
Girls, n (%)		64 (63)	29 (55)	35 (71)
Age (y), mean (SD)		15 (2)	14 (1)	17 (1)
BMI (kg/m^2^), mean (SD)		19.9 (3)	18.5 (3)	21.5 (3)
**BMI category^b^, n (%)**				
	Underweight	2 (2)	2 (4)	0 (0)
	Normal weight	84 (88)	45 (90)	39 (85)
	Overweight	10 (10)	3 (6)	7 (15)
Basal metabolic rate (kcal/day), mean (SD)		1545 (205)	1484 (186)	1610 (207)
Born in the Netherlands, n (%)		88 (92)	46 (92)	42 (91)
**Living situation, n (%)**				
	Living with both parents or caregivers	75 (78)	41 (82)	34 (74)
	Living separately^c^	18 (19)	9 (18)	9 (20)
	Living independently	3 (3)	0 (0)	3 (6)
**Educational level, n (%)**				
	Primary school	5 (5)	5 (10)	0 (0)
	Secondary school	81 (85)	44 (88)	37 (80)
	Intermediate vocational education	4 (4)	0 (0)	4 (9)
	Higher vocational and academic education	5 (5)	0 (0)	5 (11)
	Other	1 (1)	1 (2)	0 (0)
**Average exercise time (h/wk), n (%)**				
	<1	6 (6)	4 (8)	2 (4)
	1-2	16 (17)	9 (18)	7 (15)
	2-4	19 (20)	10 (20)	9 (20)
	4-6	19 (20)	10 (20)	9 (20)
	>6	36 (37)	17 (34)	19 (41)
**Physical activity^d^ (d/wk), n (%)**				
	<1	3 (3)	2 (4)	1 (2)
	1-2	11 (11)	3 (6)	8 (17)
	3-5	40 (42)	23 (46)	17 (37)
	6-7	17 (18)	8 (16)	9 (20)
	7	25 (26)	14 (28)	11 (24)
System Usability Scale, mean (SD)		72 (15)	68 (13)	67 (12)
**Number of complete dietary data collections, n (%)**				
	Two 2-hour recall days^e^	49 (48)	26 (49)	23 (47)
	Two 4-hour recall days^e^	77 (75)	39 (74)	38 (78)
	Two 24-hour recall days	91 (89)	48 (91)	43 (88)
	FFQ^f^	81 (79)	43 (81)	38 (78)

^a^Missing: 6 for BMI, BMR, born in the Netherlands, living situation, educational level, exercise time, and physical activity, and 14 for SUS.

^b^On the basis of the Schofield 10- to 18-year equation, for girls: BMR = (200.0 + [8.4 × weight]) + (465.4 × [length / 100]), and for boys: BMR = (515.3 + [16.2 × weight]) + (137.1 × [length / 100]) [34].

^c^ Living separated includes coparenting, living with the mother or father and partner, and living with the mother or father separately.

^d^Physical activity for at least an hour per week (ie, all the forms of sports or exercise in a day, including travel time to and from school).

^e^No more than one 2-hour recall or 4-hour recall missed per day.

^f^FFQ: food frequency questionnaire.

### Participant Characteristics (Phase 2)

In phase 2, interviews were held with a subsample of 24 adolescents divided between the age groups of 12 to 15 years (n=13, 54%) and 16 to 18 years (n=11, 46%), with a mean age of 15 (SD 2) years. This subsample included 18 (75%) girls, 20 (83%) participants had a healthy BMI, and 18 (75%) lived with both parents. Most (n=21, 88%) of them attended high school and 10 (42%) engaged in exercise for an average of 1 to 2 hours per week. A total of 7 (29%) participants were physically active for an average of 6 to 7 days and 7 (29%) participants for 7 days per week as shown in Table 2.

**Table 2:** General characteristics of the adolescents included in phase 2 of this study (N=24).

			Interview subsample^a^		12- to 15-year age group		16- to 18-year age group
Sample, n (%)			24 (100)		13 (54)		11 (46)
Girls, n (%)			18 (75)		9 (69)		9 (82)
Age (y), mean (SD)			15 (2)		14 (1)		17 (1)
BMI (kg/m^2^), mean (SD)			19.9 (3)		18.1 (3)		22.1 (3)
**BMI category^b^, n (%)**							
	Underweight	1 (4)		1 (8)		0 (0)	
	Normal weight	20 (83)		12 (92)		8 (73)	
	Overweight	3 (13)		0 (0)		3 (27)	
Basal metabolic rate (kcal/day), mean (SD)			1478 (156)		1417 (145)		1550 (141)
Born in the Netherlands, n (%)			21 (88)		11 (85)		10 (91)
**Living situation, n (%)**							
	Living with both parents or caregivers	18 (75)		11 (85)		7 (64)	
	Living separately^c^	4 (17)		2 (15)		2 (18)	
	Living independently	2 (8)		0 (0)		2 (18)	
**Educational level, n (%)**							
	Primary school	1 (4)		1 (8)		0 (0)	
	Secondary school	21 (88)		12 (92)		9 (82)	
	Intermediate vocational education	0 (0)		0 (0)		0 (0)	
	Higher vocational and academic education	2 (8)		0 (0)		2 (18)	
	Other	0 (0)		0 (0)		0 (0)	
**Average exercise time (h/wk), n (%)**							
	<1	0 (0)		0 (0)		0 (0)	
	1-2	10 (42)		5 (38)		5 (46)	
	2-4	8 (33)		5 (38)		3 (27)	
	4-6	6 (25)		3 (24)		3 (27)	
	>6	0 (0)		0 (0)		0 (0)	
**Physical activity^d^ (d/wk), n (%)**							
	<1	0 (0)		0 (0)		0 (0)	
	1-2	4 (17)		2 (15)		2 (19)	
	3-5	6 (25)		3 (23)		3 (27)	
	6-7	7 (29)		4 (31)		3 (27)	
	7	7 (29)		4 (31)		3 (27)	
System Usability Scale, mean (SD)			66 (25)		64 (28)		69 (69)

^a^Missing: 2 for SUS.

^b^On the basis of the Schofield 10- to 18-year equation, for girls: BMR = (200.0 + [8.4 × weight]) + (465.4 × [length / 100]), and for boys: BMR = (515.3 + [16.2 × weight]) + (137.1 × [length / 100]) [34].

^c^Living separated includes coparenting, living with the mother or father and partner, and living with the mother or father separately.

^d^Physical activity for at least an hour per week (ie, all the forms of sports or exercise in a day, including travel time to and from school).

## Discussion

This paper describes the design of the Traqq-Z study, which aims to evaluate the accuracy, usability, and user perspectives of the Traqq ecological momentary dietary assessment app in adolescents. We included 102 adolescents, with half (n=53, 52%) aged 12 to 15 years and the other half (n=49, 48%) aged 16 to 18 years, who were primarily girls and in high school. Response rates were consistently high, except for the two 2hR days. The adolescents rated the adult version of Traqq as above-average in terms of usability. A subgroup of 24 adolescents, equally divided between the age groups of 12 to 15 and 16 to 18 years, participated in semistructured interviews to further explore user preferences. Quantitative evaluation of the repeated short recall approach will reveal its potential in adolescents. Moreover, the evaluation questionnaire and semistructured interviews will yield valuable insights into adolescents’ needs and preferences for dietary assessment tools. In the final phase of the study, cocreation sessions will involve adolescents in redesigning an app tailored to their requirements.

The Traqq-Z study’s strength lies in its mixed methods approach, which comprehensively evaluates the accuracy of a novel dietary assessment approach (ie, repeated short recalls), usability of the Traqq app, and user preferences, offering insights into factors influencing accuracy, usability, and compliance [50]. An important aspect is the evaluation of the novel short recall approach, namely 2hR days and 4hR days. While repeated 2hRs effectively assess dietary intake in Dutch adults [26], they might be less suitable for adolescents due to restrictions on smartphone use during school hours. Evaluating different time intervals will help identify the optimal approach. Compliance rates suggest that 4-hour time windows are more effective in adolescents than 2-hour windows. Further analyses will show if these higher compliance rates lead to more accurate intake data.

Adolescents rated the current version of the Traqq app with above-average usability, scoring 72 out of 100 on the SUS. This score exceeds expectations for a version not yet tailored to adolescents. Comparable usability scores were found for other adolescent dietary assessment tools, such as the EaT app (69 out of 100) and the web-based myfood24 (74 out of 100) [19,21]. These findings indicate that the current version of the Traqq app serves as a solid starting point. Analyses of the evaluation questionnaire and semistructured interviews will pinpoint areas for improvement and customization opportunities for the app.

This research also facilitates tailoring of the app to adolescents through a user-centered design approach. Involving target users in the redesign process ensures that the adapted app meets their needs and preferences. Cocreation sessions allow adolescents to refine the tool, which is crucial for identifying and addressing potential issues such as difficulties in estimating food intake. Integrating insights from both quantitative and qualitative evaluations into the redesigned Traqq app aims to improve usability, compliance, and accuracy [51]. Validation studies comparing the current and adapted versions with reference methods will follow to confirm these adaptations.

Moreover, while more than 3 recall days per method would likely yield more accurate dietary intake estimates [52], 2 recall days were selected due to their acceptable agreement with the FFQ for regularly consumed foods and nutrients [53], as well as to minimize participant burden. Altogether, this approach offers valuable insights into the feasibility and optimal recall period of the repeated short recall approach for assessing dietary intake among adolescents.

To conclude, the most important asset of the Traqq-Z study lies in its comprehensive study design. The results include both quantitative data, including dietary intake data acquired through two 2hR days, two 4hR days, 2 interviewer-administered 24hRs, and one FFQ and an evaluation questionnaire. In addition, qualitative data from semistructured interviews will provide further insight into the needs and preferences of adolescents on dietary assessment tools. Cocreation sessions involve adolescents in the redesign process of a tailored version of the Traqq app. An extensive evaluation of the redesigned dietary assessment app is crucial to ensure usability and accuracy of the method, not just against traditional methods, but against objective measures as well.

## Appendix

Multimedia Appendix 1 Interview guide—user perspectives.

## Abbreviations

2hR: 2-hour recall
4hR: 4-hour recall
24hR: 24-hour recall
COM-B: capability, opportunity, motivation-behavior
FFQ: food frequency questionnaire
SUS: System Usability Scale
