# Supplementary material for: Evaluation of the Accuracy, Usability, and User Perspectives of the Ecological Momentary Dietary Assessment App Traqq Among Dutch Adolescents: Protocol for a Mixed Methods Study
Source: JMIR Res Protoc. 2025 Nov 11;14:e70194. doi: 10.2196/70194 (PMC12648135; doi:10.2196/70194)
Supplement: Multimedia Appendix 1 [file resprot_v14i1e70194_app1.docx]

**Interview Guide - User perspectives.**

1. Why did you participate in the study?
   1. How did you come into contact with the study?
   2. What were your thoughts on the study?
   3. Would you participate in a study again?
   4. What did you learn during the study?
   5. What did you think of the infographic you received as a thank-you gift?
2. Can you describe your experience using the app?
   1. What worked well in the app?
   2. What made it difficult to use the app? Were there any activities that got in the way of using it?
      1. If yes, when?
   3. On average, how much time did you spend using the app (differentiate between 2-hour and 4-hour intervals)?
   4. Would you use this app in the future to track your food and drink intake?
      1. Why or why not?
   5. Are there any features you feel are missing from the app? Do you have suggestions or ideas for improvement?
3. Did your eating or drinking habits change while using the app?
   1. If yes, is this change still present?
   2. What do you think about that?
   3. Did using the app make you more aware of what you eat and drink?
      1. What made you more aware? (Change in eating habits?)
      2. Is there anything else you would like to know about your diet? (Would you like more feedback?)

***Think aloud exercise***

*Let’s take a look at the app, starting with the design.*

1. What are your first impressions? What are your preferences regarding the app’s design?
   1. What makes the app fun or interesting to use?
   2. What could make the app easier to use?
   3. What do you think of the homepage?
   4. What do you think of the color scheme?
   5. What would help maintain your attention? (e.g., interaction, feedback, notifications, speed, clarity, logic, visuals, images?)
   6. Do you have any tips or ideas to make the app more engaging or fun? What would motivate you to respond to notifications or use the app to track your diet? (Ideas from other apps you’ve used?)

*You used the app to track your* *food and drink intake. Could you open the app now and enter what you had for lunch today?*

1. How was the experience of entering what you ate and drank?
   1. Could you easily find all the food and drinks you consumed?
      1. If not, what issues did you encounter? Can you give an example?
      2. If yes, do you have ideas to make this even easier?
      3. On average, how long did it take you to search for the foods you ate?
         1. How could this process be made easier? Any ideas?
         2. Did you use the ‘My dish’ function?
2. How did you find estimating the amount of food and drink you consumed?
   1. Did you receive help with this?
      1. If yes, was it during a specific meal?
      2. If yes, what could help you avoid needing assistance in the future?
   2. What method did you use to indicate quantities, grams or standard measures (e.g., a bowl of yogurt)?
      1. Why did you choose that option?
3. For how many consecutive days would you be willing to use the app to track your intake?

How many non-consecutive/random days would you be willing to track?

1. How did you find receiving an invitation every 2 hours to enter what you ate and drank?
   1. What worked well, and what didn’t go so well when responding to the notifications?
2. How did you find receiving an invitation every 4 hours to enter what you ate and drank?
   1. What worked well, and what didn’t go so well when responding to the notifications?
3. Were there specific time periods that were more difficult to track?
   1. If yes, why was it harder during those times?
4. If you could choose your own interval to receive notifications, what would be your preferred time interval?
   1. Why would you prefer this time interval?
      (If longer than 4 hours: Do you think you would still remember what you had eaten and drunk?)
5. What did you think of the time you had to respond to the notification? (Reminder: the response window was 1.5 hours.)
   1. What would be your ideal duration for the notification window?
   2. What do you think about reminders that signal the start or end of a time period?

*Is there anything else you would like to add or something we haven’t discussed that you think is important to share?*
